# Metabolic activity and community structure of prokaryotes associated with particles in the twilight zone of the South China Sea

**DOI:** 10.3389/fmicb.2022.1056860

**Published:** 2022-12-06

**Authors:** Hao Liu, Fangzhou Wang, Hongbin Liu, Hongmei Jing

**Affiliations:** ^1^CAS Key Lab for Experimental Study Under Deep-sea Extreme Conditions, Institute of Deep-sea Science and Engineering, Chinese Academy of Sciences, Sanya, China; ^2^University of Chinese Academy of Sciences, Beijing, China; ^3^Department of Ocean Science, The Hong Kong University of Science and Technology, Kowloon, Hong Kong SAR, China; ^4^HKUST-CAS Sanya Joint Laboratory of Marine Science Research, Chinese Academy of Sciences, Sanya, China; ^5^Southern Marine Science and Engineering Guangdong Laboratory (Zhuhai), Zhuhai, China

**Keywords:** twilight zone, particulate organic carbon, size fractions, microbial metabolic activity, South China Sea

## Abstract

The twilight zone is an important depth of the ocean where particulate organic matter (POM) remineralization takes place, and prokaryotes contribute to more than 70% of the estimated remineralization. However, little is known about the microbial community and metabolic activity associated with different particles in the twilight zone. The composition and distribution of particle-attached prokaryotes in the twilight zone of the South China Sea (SCS) were investigated using high-throughput sequencing and quantitative PCR, together with the Biolog Ecoplate™ microplates culture to analyze the microbial metabolic activity. We found that α- and γ-Proteobacteria dominating at the lower and upper boundary of the twilight zone, respectively; *Methanosarcinales* and *Halobacteriales* of the Euyarchaeota occupied in the larger particles at the upper boundary. Similar microbial community existed between euphotic layer and the upper boundary. Higher amount of shared Operational Taxonomic Units (OTUs) in the larger particles along the water depths, might be due to the fast sinking and major contribution of carbon flux of the larger particles from the euphotic layer. In addition to polymers as the major carbon source, carbohydrates and amino acids were preferentially used by microbial community at the upper and lower boundary, respectively. This could potentially be attributed to the metabolic capabilities of attached microbial groups in different particles, and reflected the initial preference of the carbon source by the natural microbes in the twilight zone as well. The microbial structure and carbon metabolic profiles could be complemented with metatranscriptomic analysis in future studies to augment the understanding of the complex carbon cycling pathways in the twilight zone.

## Introduction

Twilight zone with water depths of 200 ~ 800 m is characterized with weak light and unstable carbon and energy supply (Buesseler et al., 2007). Most particulate organic carbon (POC) generated from the euphotic zone is degraded and remineralized while sinking through the water column (Buesseler et al., 2007), particularly in the twilight zone, leading to the fact that only 0.1 ~ 0.3% POC was buried in the seafloor sediments (Arístegui et al., 2005, 2009). Prokaryotes are responsible for about 70–90% of the POC being decomposed and remineralized in the twilight zone (Arístegui et al., 2009; Jiao et al., 2010), which subsequently determining the carbon flux and carbon sequestration to the deep waters, influencing the efficiency of the biological and microbial carbon pumps (Jiao et al., 2010).

Different types of POC existed in the seawater, including suspended sediment particles, phytoplankton debris, living plankton, zooplankton fecal materials, aggregates, marine snow, transparent polymeric particles, colloidal particles and so on (Azam and Malfatti, 2007; Dang and Lovell, 2016). Microbes are involved in different dynamic processes of POC, such as aggregations, depolymerization, remineralization and sedimentation of particles, continuously occuring in the twilight zone (Volk and Hoffert, 1985). The rate of converting sinking POC into CO_2_ by heterotrophic prokaryotes controls the carbon storage in the ocean (Jiao et al., 2014). Particle-attached microbes are usually more diverse and less responsive to environmental variables than free-living bacteria (Yung et al., 2016; Zhang et al., 2020), because of their higher hydrolytic activity of POC utilization (Ivancic et al., 2018). Particle size is an important parameter affecting the microbial composition and activity on the particle (LaMontagne et al., 2003; Eloe et al., 2011). Therefore, molecular ecological studies on microbes attached to particles of different sizes will help to track their population shifts and potential functions in the POC transformation in different depths of the twilight zone.

The Biolog EcoPlates^™^ method, relying on the microbial capability to use various carbon sources, is a simple and sensitive way to reveal the functional diversity of microbial community. It has been widely used for assessing the metabolic activity of microbes in various environments, such as wetland (Teng et al., 2019), sediment (Lopes et al., 2016; Sinha et al., 2019) and reservoir (Zhou et al., 2019). By far, the POM utilization by microbial groups in the twilight zone has been studied by proteomic and metaproteomic approaches (Dong et al., 2010; Kong et al., 2021a, 2021b), but there is still a lack of direct evidence of carbon source utilization by different microbial groups in different depths of the twilight zone. Carbon sources of POC have been reported to vary with different size-fractions and water depths (Azam et al., 1992; Simon et al., 2002; Steinberg et al., 2008). Previous studies using Biolog Phenotype MicroArrays technology demonstrated that different bacterial strains could use different carbon sources (Techtmann et al., 2015). Therefore, culture-dependent EcoPlates cultivation together with culture-independent molecular study would help to get a quick glimpse of the shift of ecological roles of microbial communities from the euphotic zone to the twilight zone.

In our study, particles of three different size-fractions were collected from the upper and lower boundary of the twilight zone in the SCS, compared with those from the euphotic layer. Diversity and composition of the microbial community associated with particles were studied with high-throughput sequencing and quantitative PCR. Biolog EcoPlates^™^ method was applied as well to investigate the carbon metabolic capability of microbes. The objective of this study was to better understand the shifts and connectivity in the diversity and specific carbon metabolic capabilities of microbial communities in the twilight zone.

## Materials and methods

### Sample collection

Seawater samples were collected (16°43′55.8” N, 110°27′40.4″ E) in the SCS during cruise TS2-3-2 in Feb. 2021. Niskin bottles were used to collect water samples from three discrete depths (i.e., 0 m, 200 m and 800 m), and about 2 l seawater were prefiltered through 200 μm mesh, and then sequentially through 50 μm, 10 μm mesh and then 1 μm pore-size polycarbonate filter (47 mm, EMD Millipore, Billerica, MA, United States). All the filters were then flash frozen and stored at −80°C until DNA extraction in the laboratory.

### Measurement of environmental variables

The *in situ* environmental parameters (temperature, salinity, depth) were recorded with a conductivity-temperature-depth (CTD, Sea-Bird Electronics). The concentrations of inorganic nutrients (nitrate, nitrite, ammonia, phosphate and silicate) were analyzed with an auto-analyzer (QuAAtro, Blue Tech Co., Ltd., Tokyo, Japan). To determine the cell abundances of *Synechococcus*, *Prochlorococcus*, picoeukaryote and heterotrophic bacteria, 1.80 ml seawater was fixed with seawater buffered 0.5% ~ 1% paraformaldehyde and stored at −80°C.

Seawater samples were filtered through pre-combusted (450°C for 4 h) glass fiber filters (GF/F; Whatman), with a nominal pore size of 0.7 μm. The filters were kept frozen at −20°C until analyzed in the laboratory. The filters were fumed overnight with HCl using the vapor method (Turnewitsch et al., 2007) to remove carbonates and then dried at 60°C for 24 h. POC was quantified with an elemental analyzer (NA-1500; Fisons Instruments), using acetanilide (Thermo Electron) as a standard. DOC was determined by high temperature catalytic oxidation method and determined using a Shimadzu TOC-VCPH analyzer (Ogawa et al., 1999).

### Microbial metabolic activity analyses

Water samples were collected from the euphotic layer (0 m) and the upper (200 m) and lower boundary (800 m) of the twilight zone in the SCS, then filtered sequentially through 200, 50, 10 μm mesh and 1 μm pore-size polycarbonate filters. About 150 μl of each filtrate was added into every hole of the Biolog Ecoplate™ microplates. The ecological plate from samples collected from 0 m was cultured at room temperature (~25°C), those from 200 m was cultured at room temperature without light (one layer of black plastic bag), and those from 800 m was cultured at ~4°C without light (two layers of black plastic bag). The absorbance of each well was measured at 590 nm and 750 nm every 24 h for a continuous cultivation of totally 52 days. The difference of absorbance value was used to characterize the color change of the Biolog Ecoplate^™^ microplates by eliminating the turbidity change caused by fungi at 750 nm. The average well color development (AWCD) was calculated to determine the utilization of carbon sources and metabolism characteristics.


(1) $$\mathrm{AWCD}=\frac{\sum R-C}{n}$$

In equation (1), R is the absorbance of each well, C is the absorbance of the control well and n is the number of substrates present in the particular category (Feigl et al., 2017). Meanwhile, the indexes of Richness (R), Simpson (D) and Shannon (H) were calculated to reflect the metabolic function diversity of microbial community. Richness index refers the number of oxidized substrates. It was computed as the total of the cells’OD_i_ value, which was at least 0.5 after incubation (Farkas et al., 2020). OD_i_ is the corrected OD value of each individual well, at two consecutive measurements at two different measurement times for t_n_ and t_n + 1_.

Simpson index (D) was computed by the following equation (2):


(2) $$D=-\ln\overset{N}{\underset{i=1}{\sum }}{\left(Pi\right)}^{2}$$

where N is the number of substrates; P_i_ is the relative color development of the well over the total color development of each well of a plate, as shown in the equation (3) (Tam et al., 2001).


(3) $$\mathrm{Pi}=\frac{\mathrm{ODi}}{\sum_{i=1}^{N}ODi}$$

Shannon index (H) was calculated according to the following equation (4):

(4) $$H=-\overset{N}{\underset{i=1}{\sum }}Pi.\ln Pi$$

where N and P_i_ are the same as in the Simpson index calculation (Moore, 2013).

### DNA extraction, PCR amplification and sequencing

Total genomic DNAs from 50, 10 and 1 μm pore-size polycarbonate filters were extracted using a PureLink Genomic DNA Mini Kit (Invitrogen, Thermo Fisher Scientific, Corp., Carlsbad, CA, United States). The DNA was amplified *via* PCR using universal prokaryotic primers: Pro341F (5’-CCTACGGGNBGCASCAG-3′) and Pro805R (5’-GACTACNVGGGTATCTAATCC-3′; Takahashi et al., 2014), targeting the V3-V4 region of the 16S rRNA gene. These primers were tagged with a 6 bp barcode for differentiation of amplicons in the pools of all samples multiplexed for Illumina. PCR amplification was carried out in triplicate using the BIO-RAD C1000 Touch^™^ Thermal Cycler PCR System in a 20 μl PCR reaction mix, containing 2.0 μl 10 × PCR-MgCl_2_ buffer, 0.7 μl 2.5 mM dNTPs, 0.7 μl MgCl_2_, 0.8 μl forward primer, 0.8 μl reverse primer, 0.2 μl Platinum® TaqDNA ploymerase, 2.5 μl template DNA and 12.3 μl ddH_2_O. Thermal cycling was performed at 95°C for 3 min, followed by 33 cycles at 95°C for 0.5 min, 55°C for 45 s, 72°C for 30 s, and a final extension at 72°C for 8 min. Double-distilled water was as a negative control. Amplification and paired end sequencing of the amplicons were then performed with an Illumina HiSeq PE250 sequencer (Novogene Co., Ltd.).^1^

### Quantitative PCR

The abundance of the 16S rRNA gene was quantified using the StepOnePlus qPCR system (Applied Biosystems Inc., Carlsbad, CA, United States). Each qPCR reaction comprised 10 μl 2 × SYBR^®^ Premix Ex Taq^™^II (Takara Bio. Inc., Shiga, Japan), 0.3 μM Uni340F (5’-CCTACGGGRBGCASCAG-3′)/Uni806R (5’-GGACTACNNGGGTATCTAAT-3′) primer (Takai and Horikoshi, 2000), 2 μl DNA as the template, 0.4 μl ROX reference dye, and water to a total of 20 μl. Quantitative PCR reactions and calibrations were performed as reported before (Takai and Horikoshi, 2000). Triplicate qPCR reactions were performed for each sample with efficiencies of ~92.4%, and the gene copy number was normalized to the quantity of the gene (Table 1).

**Table 1:** Sequencing information and diversity parameters of the 16S rRNA gene in this study.

Depth (m)	Size-fraction (μm)	Original Reads	Quality Reads	No. of OTUs	Simpson (97%)	Shannon (97%)	Chao1 (97%)	Coverage (97%)
0	200–50	65,536	29,679	348	0.96	5.90	362.86	0.99
	50–10	60,805	27,250	331	0.95	5.70	345.20	0.99
	10–1	65,179	30,423	237	0.95	5.39	250.12	0.99
200	200–50	64,420	34,308	406	0.97	6.56	418.12	0.99
	50–10	64,420	31,230	343	0.95	6.38	349.75	0.99
	10–1	62,896	33,444	175	0.92	4.63	183.90	0.99
800	200–50	64,471	30,769	167	0.77	3.61	170.24	0.99
	50–10	61,009	26,522	207	0.90	4.91	210.50	0.99
	10–1	65,536	32,051	197	0.89	4.40	201.35	0.99

### Bioinformatics analysis

After sequencing, barcoded and low-quality sequences were removed using QIIME with default parameters (Caporaso et al., 2010). Chimeras were detected and removed with UCHIME against the SILVA database release 128 (Pruesse et al., 2007),^2^ and reads presented as a single copy (i.e., singletons) were removed manually. The remaining reads were then clustered into Operational Taxonomic Units (OTUs) at 97% sequence similarity. Taxonomy assignment of OTUs that were not affiliated with prokaryotes, as determined from the SILVA database release 128, were further removed (Caporaso et al., 2010). A filtered OTUs table of each sample was generated with QIIME 1.9.1. The richness estimator (Chao1), diversity (Shannon and Simpson), and Good’s coverage were then calculated with 97% sequence similarity as cutoff value. For the prediction of functional and metabolic profiles of the bacterial community based on the 16S rRNA gene sequences, the recently developed open-source R package Tax4Fun (Asshauer et al., 2015) was used with the short reads mode disabled along with the SILVA database 128 as required.

### Statistical analysis

The non-linear multidimensional scaling (nMDS) based on the Bray–Curtis similarity index was calculated with PRIMER 5 (Plymouth Marine Laboratory, West Hoe, Plymouth, United Kingdom; Clarke and Warwick, 1994) to show the similarity among different samples. An analysis of similarities (ANOSIM) based on the OTU relative abundance was conducted with Paleontological Statistics (PAST) version 3 (Hammer et al., 2001) to test whether there was a significant difference in the microbial community structure and potential metabolic function among the different samples. Values of *p* < 0.05 and *p* < 0.01 were considered to indicate different levels of statistical significance. The similarity percentage analysis (SIMPER) test was used to reveal organisms that responsible for the dissimilarity observed in community composition among different samples. A redundancy analysis (RDA) was performed with CANOCO V5.0 to identify a possible differentiation of the communities under the constraint of environmental factors. The statistical significance of an explanatory variable added in the course of forward selection was tested with the Monte Carlo permutation test (9,999 permutations, *p* < 0.05). The phylogenetic group data were Hellinger transformed, environmental variables were logarithm transformed, and the effects of collinearity (VIF > 10) were removed.

## Results

### Hydrographic conditions

**Figure 1:**
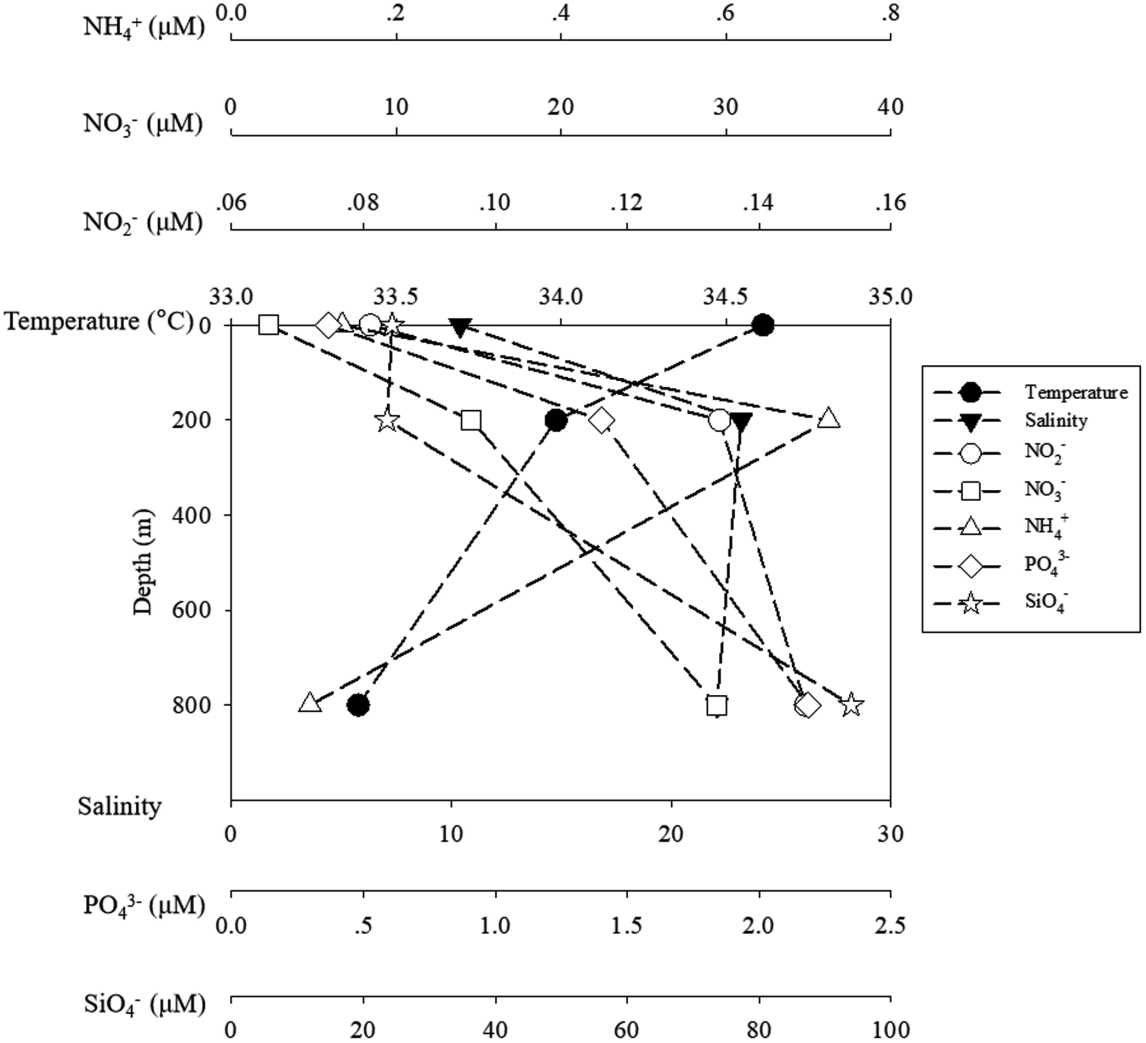
The temperature and salinity and inorganic nutrients of ambient water samples. The black symbols of circle, square stand for temperature and salinity, while the blank symbols of circle, square, triangle, diamond and star stand for NO_2_^−^, NO_3_^−^, NH_4_^+^, PO_4_^3−^ and SiO_4_^−^, respectively.

Seawater temperature decreased sharply from 24.17°C at the surface to 5.75°C at the lower boundary of the twilight zone (800 m). Salinity increased from 0 to 200 m from 33.69 to 34.54‰ and remained constant from 200 to 800 m. Generally, the concentrations of ambient inorganic nutrients (NO_2_^−^, NO_3_^−^, and PO_4_^3−^) increased with depths, and reached the maximum at 800 m, but for NH_4_^+^, the highest and lowest concentrations were detected at 200 m (0.72 μM) and 800 m (0.095 μM), respectively (Figure 1).

### Microbial community diversity and composition

**Figure 2:**
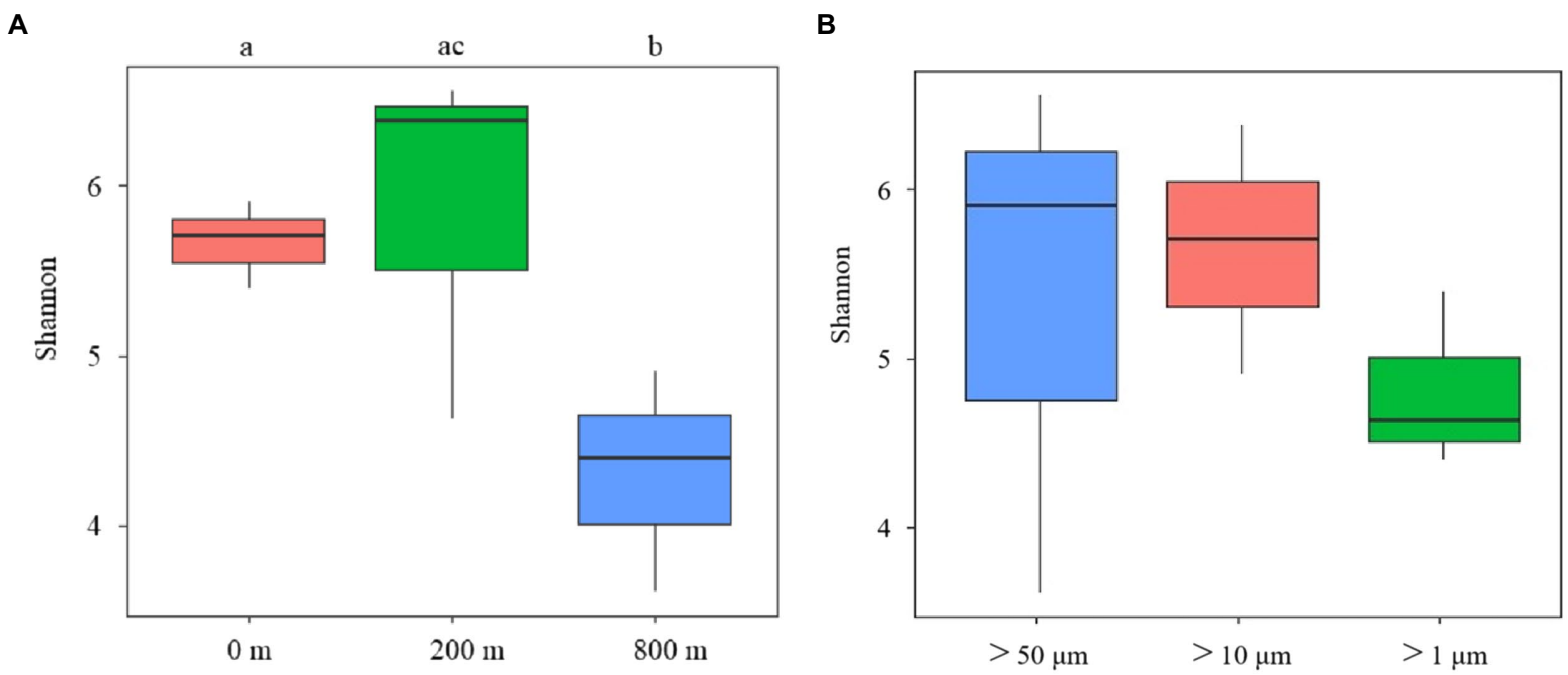
Diversity analysis of microbial communities among samples collected from different depths **(A)** and different size-fraction particles **(B)** of the South China Sea based on Shannon index (ab: *p* < 0.05, bc: *p* < 0.01).

**Figure 3:**
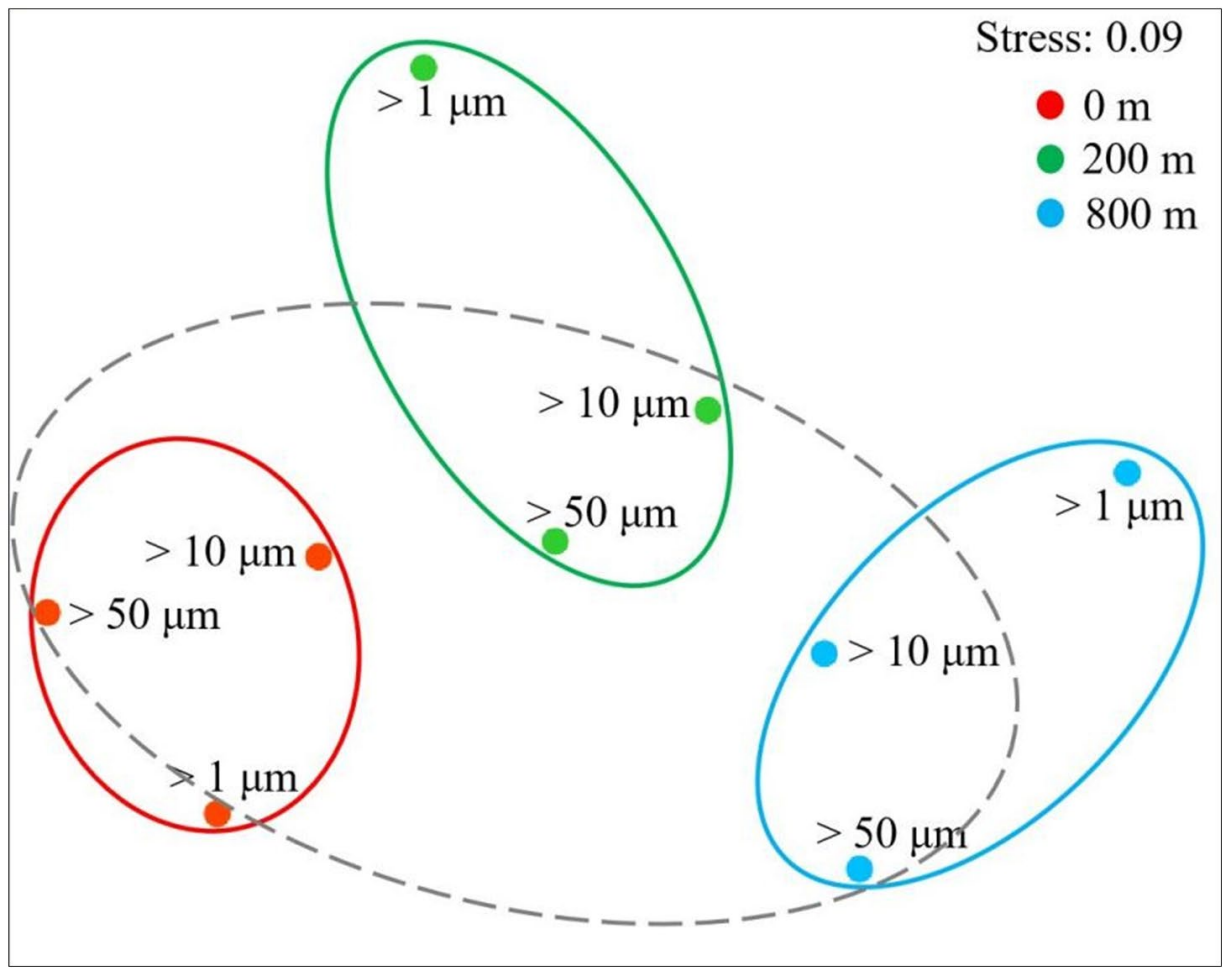
Non-linear multidimensional scaling (nMDS) analysis of microbial communities of different size-fraction particles at different water depths based on Bray-curtis distances.

In terms of Shannon diversity of the prokaryotic communities, significant differences were found between 0 and 800 m (*p* < 0.05), and between 200 and 800 m (*p* < 0.01; Figure 2A), however no significant difference existed between different size-fraction particles among all depths (Figure 2B). NMDS plot demonstrated a distinct distribution of OTUs among the three water depths (*p* < 0.05); and those from particles of >50 and > 10 μm were relatively closely distributed (Figure 3).

**Figure 4:**
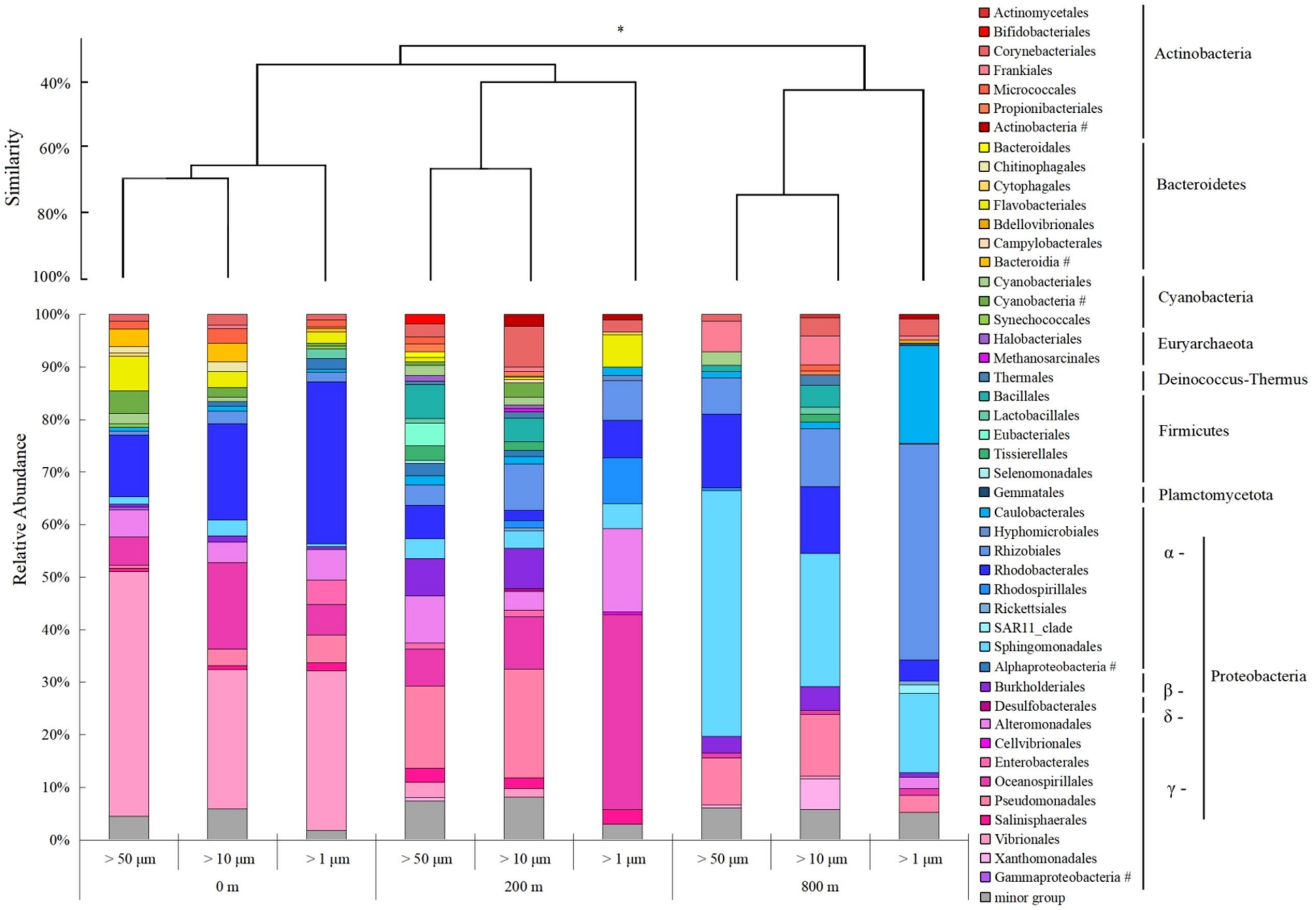
Microbial community structure of different size-fraction particles in different water depths at the order level with cluster at the phylum level. *p < 0.05, # unclassified.

Microbial community structures varied with depths, and those at 800 m were more distinct from the other two depths (ANOSIM, *p* < 0.05; Figure 4). Consistent with the lower Shannon diversity of microbial community observed at 800 m (Figure 2A), much less microbial groups at the order level were revealed from 800 m. Proteobacteria predominate in all the samples, and γ-Proteobacteria dominate at 0 and 200 m, but was shifted to α-Proteobacteria at 800 m. For different particles, the relative abundance of γ-Proteobacteria increased at 200 m, except for particles of >10 μm, but only accounted for small proportions at 800 m. With the decrease of size-fraction, a decrease of γ-Proteobacteria proportion and an increase of α-Proteobacteria proportion were observed at both layers of the twilight zone; and proportions of *Rhodobacterales* decreased as well, with *Sphingomonadales* and *Rhodobacterales* dominant in the particles of 50–200 μm at the lower boundary of the twilight zone (Figure 4). *Methanosarcinales* and *Halobacteriales* belonged to Euyarchaeota were only found with larger particles (>50 and 10 μm) at the upper boundary of the twilight zone. Based on qPCR analysis, the 16S rRNA gene abundance of prokaryotes decreased from 200 to 800 m. At different depths, the gene abundance of prokaryotes was always the highest in particles of >1 μm. As for particles of >50 and 10 μm, significantly higher gene abundance of prokaryotes was appeared at 200 m than at 0 and 800 m (*p* < 0.01; Supplementary Figure S1). The cell abundances of *Synechococcus* and *Prochlorococcus* with different size particles were always higher at 0 m and decreased with depths, and their cell abundance with larger particles (> 50 and 10 μm) was generally consistently higher than small fraction of particles with three depths (Supplementary Figure S1).

RDA analysis revealed that the first and second axes together contributed 58.94% to the total variance of the whole communities (Supplementary Figure S2). Among the tested environmental factors, temperature (*p* < 0.01), salinity (*p* < 0 0.05) and depth (*p* < 0.01) were statistically significant contributing to the variation of prokaryotic communities (Supplementary Figure S2).

### Composition of the core components

Venn diagrams showed the overlapping of OTUs among different samples, and the shared OTUs was defined as core components (Supplementary Figure S3A). Among different sizes, the number of core OTUs was the highest at 0 m, and decreased with water depth. The core components comprised mainly of α- and γ-Proteobacteria, and the proportion of Actinobacteria increased with depths, and Cyanobacteria only appeared at 0 and 200 m (Supplementary Figure S3B). Among different depths, the number of core OTUs was the highest in the fraction of >50 μm, and decreased with the decrease of size fractions (Supplementary Figure S3B). Core components were dominated by α- and γ-Proteobacteria, and the latter increased with the decrease of size fractions. The relative abundance of shared OTUs between the fractions of >50 and > 10 μm was highest in either >50 or > 10 μm fractions at all depths, expect for 800 m (between >50 and > 1 μm fractions, Supplementary Table S1), and the relative abundance of shared OTUs between 0 and 200 m was highest in all size fractions, expect for >1 μm fraction (between 200 and 800 m, Supplementary Table S2).

### Prokaryotic metabolic activity and potential function

**Figure 5:**
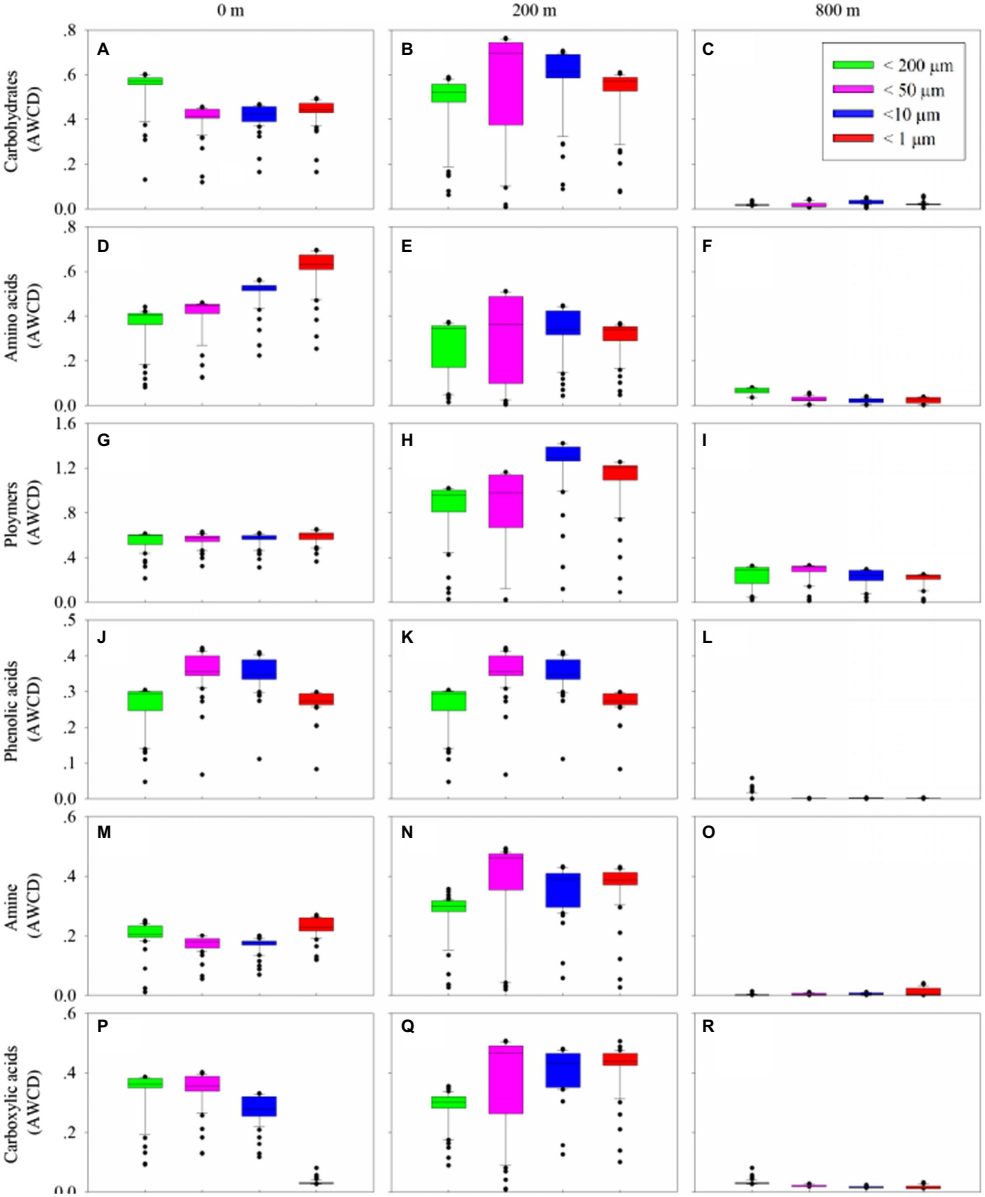
Box plots of utilization capability of the six major carbon groups, carbohydrates **(A–C)**, amino acids **(D–F)**, polymers **(G–I)**, phenolic acids **(J–L)** and amine **(M–O)**, carboxylic acids **(P–R)**, by microbes on different size-fraction particles at different water depths.

Biolog Ecoplate™ microplates contained 31 different carbon sources belonging to 6 carbon categories, including carbohydrates, polymers, carboxylic /amides/amino acids and amines, to show the microbial capability of carbon utilization. Based on AWCD, the utilization of different carbon categories by microbes mainly occurred in samples collected from 0 and 200 m (Figure 5). At 0 m, amino/ carboxylic acids were mainly used by those with smaller fractions (< 10 μm), while carbohydrates were mainly used by those with larger fractions (< 200 μm; Figures 5A,D,G,J,M,P). At 200 m, polymers were mainly used by those with smaller fractions (< 10 μm), and the remaining categories were mainly used by microbes with fractions of <50 and 10 μm (Figures 5B,E,H,K,N,Q). At 800 m, amino acid and carboxylic acids were mainly used by those with fractions of <200 μm (Figures 5C,F,J,L,O,R), whilst polymers were mainly used by those from larger fractions (< 50 and 200 μm; Figure 5I).

**Figure 6:**
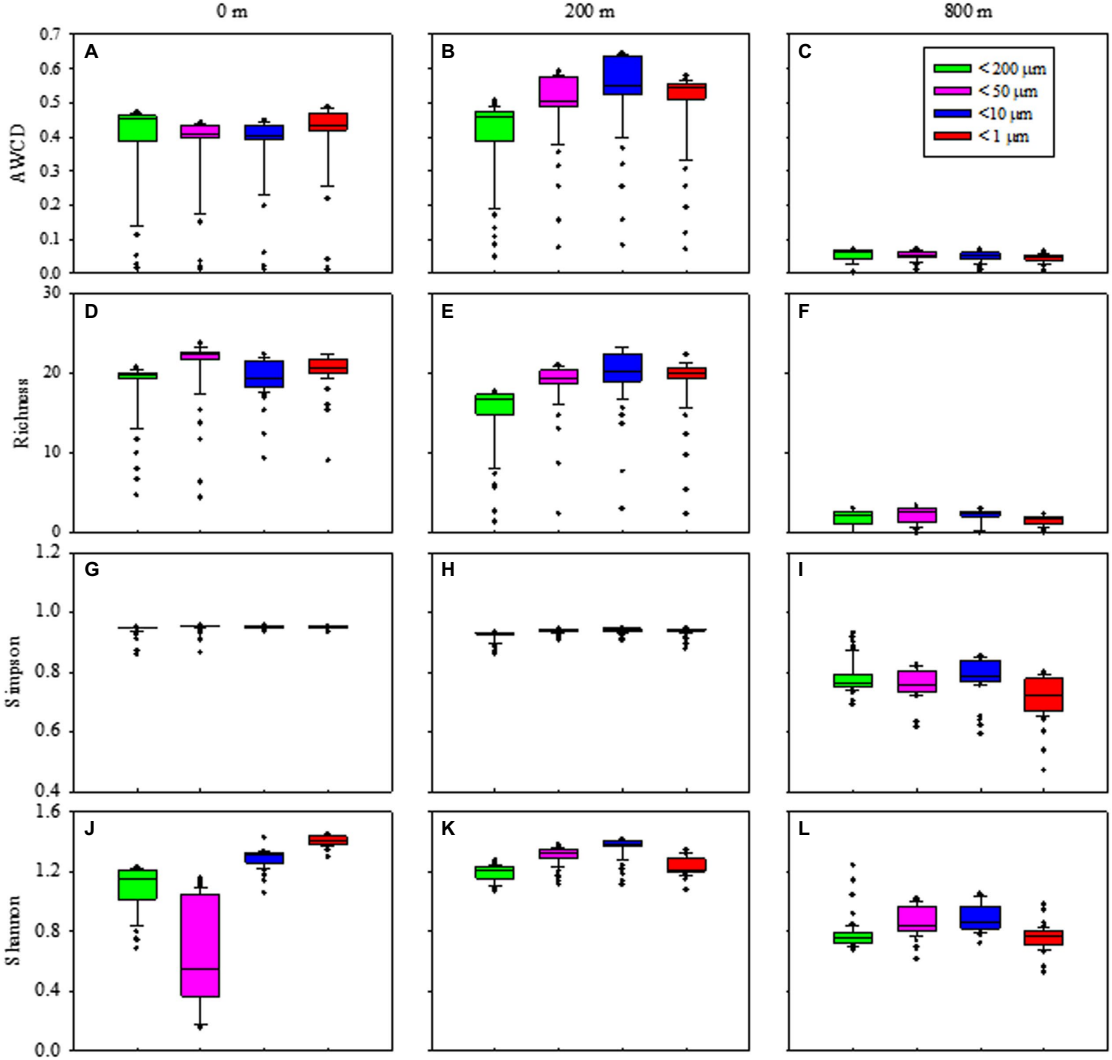
Box plots of the microbial metabolic activity of AWCD **(A–C)**, Richness index **(D–F)**, Simpson index **(H–J)**, and Shannon index **(K–M)** associated with different size-fraction particles at different water depths.

Metabolic activity reflected by AWCD demonstrated that microbes first entered the exponential growth period and then the stable period during cultivation (Supplementary Figures S6A–C). Metabolic diversity (AWCD, Richness, Simpson and Shannon) were significantly higher at 0 and 200 m than at 800 m (*p* < 0.05; Figure 6). Microbial community entered the stable period on day 15 for samples from 0 m (Supplementary Figure S6A), while on respective day 23 and 27 for samples from 200 and 800 m (Supplementary Figures S6B,C). The highest metabolic activity during the exponential growth period from smaller (< 10 μm) and larger (< 200 μm) fractions was appeared at 200 m (Supplementary Figure S6B) and 800 m (Supplementary Figure S6C), respectively. During the whole cultivation period, metabolic diversity (Simpson and Shannon) increased from 0 to 200 m at the exponential growth period (Supplementary Figures S6G,H,J,K), while showed a first decrease and then stable at 800 m for samples with larger sizes (< 200 μm; Supplementary Figures S6I,L). Metabolic diversity for particles of <200 μm increased gradually and almost reached the same level as those of smaller sizes at 0 and 200 m (Figure 6).

In addition, KEGG categories were assigned to the selected OTUs subsets belonging to the core components among all samples. of Generally, five different metabolic categories were identified, and they were significantly different between euphotic layer and the twilight zone (200 and 800 m; *p* < 0.05; Supplementary Figure S4). Carbohydrate metabolism was the most abundant functional category, followed by clycan biosynthesis and metabolism; both of which were more abundant at the twilight zone.

## Discussion

### Microbial population shift

In this study, significantly lower prokaryotic gene abundance in all POM size-fractions found at the lower boundary than at the upper boundary of the twilight zone. This is possibly because POM was more refractory at the lower boundary in the twilight zone (Pakulski and Benner, 1994; Mayor et al., 2011). Prokaryotic gene abundance associated with 10–50 μm particles was significantly lower than those with smaller particles at the euphotic layer (*p* < 0.01), but no significant difference found in the twilight zone. This possibly because that 10–50 μm particles at surface were mostly living cells (e.g., phytoplankton; Supplementary Figure S1) with less bacteria colonizing on them.

Microbial diversity at the lower boundary of the twilight zone was significantly different from others, maybe due to the continuous connection of the two depths from the surface to the upper boundary of the twilight zone. A clear shift of γ-Proteobacteria in the euphotic layer to α-Proteobacteria at the lower boundary in the twilight zone was observed, could be attributed to the temperature effect as demonstrated by the RDA analysis. Previous study showed that in the SCS, γ-Proteobacteria were distributed mainly in the upper 200 m, while some of α-Proteobacteria were distributed between 0 and 1,000 m depths (Chen et al., 2019), indicating α-Proteobacteria could adapt to lower temperature. Meanwhile, in the twilight zone of Northwest Pacific Ocean, the dominant group could shift from *Alteromonadales* in the high-temperature layers to *Enterobacterales* in the lower-temperature layers (Kong et al., 2021a). This further proved that temperature was the main environmental factor modulating the composition and metabolic activity of the microbial groups involved in the remineralization of POC in the twilight zone (Kong et al., 2021a).

### Core microbial components

For the core microbial community from different depths, more shared OTUs were found between euphotic layer and the upper boundary of the twilight zone. Suspended particles in euphotic waters were composed primarily of labile phytoplanktonic materials with similar pigment, lipid, and amino acid composition (Sheridan et al., 2002), and they became less degradable with more complicated compositions once sinking out of the euphotic zone (Dong et al., 2010), thus supporting the growth of different microbial groups within and below euphotic layer. α- and γ-Proteobacteria dominated, respectively, at the lower and the upper boundary of the twilight zone. This might due to the optimal conditions required by the two phyla, since the latter could attach to particles to avoid the nutrient-depleted conditions in the surrounding waters (Acinas et al., 1999) and easily assimilated organic carbon sources for rapid growth capacity (Pinhassi and Berman, 2003), but were selectively outcompeted by the former in the deep waters in the SCS (Chen et al., 2019).

As for different size particles, more shared OUTs appeared in the larger particles (> 50 and 10 μm) for three depths. This indicated that larger particles could rapidly sink from the euphotic layer to the twilight zone, and therefore the attached microbial community was more stable. Larger particles are usually rich in organic carbon, and they sink rapidly in the form of marine snow (Goldthwait et al., 2004). The export flux of POC in the euphotic and twilight zones in the northern SCS has been studied with ^228^Th/^228^Ra disequilibrium method (lv, 2005), which showed that larger particles contributed more to the carbon flux and sequestration to the deep sea, and small particulate matters were remineralized in the twilight zone (Buesseler et al., 2007). It was already known that particle sizes could affect the sedimentation of particles, and should be considered to elucidate the process of POC remineralization in the twilight zone (Iversen and Ploug, 2013; Cavan et al., 2019).

### Carbon source utilization and ecological significance

Microorganisms play a key role in substrate decomposition and remineralization processes in the twilight zone, therefore understanding how microorganisms utilize substrates and gain energy would be important to elucidate the biogeochemical processes in the twilight zone. Though the natural carbon sources could be much more complicated than just 31 carbon sources contained in the Biolog Ecoplate™ microplates, this method would at least reflect to certain extent the ability of microbial community to use different carbon sources and their metabolic activities. In this study, the microbial metabolic activity, reflected by AWCD values, at the upper boundary was significantly different from those at the lower boundary of the twilight zone. This difference might be caused by significantly different community compositions between the two layers, and also reflect the distinction of organic detritus at these two boundaries. The majority of sinking organic matter in the twilight zone was in the form of marine snow, faecal pellets and other particles of detritus, and might have been previously ingested and reworked multiple times by zooplankton with selectively absorbance of the most labile and nutritious dietary compounds (Mayor et al., 2011), leading to the increase of the relative proportions of refractory polysaccharides in detritus at the lower boundary of the twilight zone (Pakulski and Benner, 1994).

It was clear that the preferentially utilized organic matters during microplate incubation were those could be easily degraded, because these compounds were considered to be the largest bioavailable source of carbon in the water column (Lee et al., 2000; He et al., 2015). At the upper boundary of the twilight zone, polymers and carbohydrates were used preferentially, followed by amino acids. This might be due to the high concentration of polymers and carbohydrates at the upper boundary of the twilight zone which were used by *Alteromonadales* and *Pseudomonadales* as carbon sources (He et al., 2015; Sinha et al., 2019). *Alteromonadales* could secrete a series of extracellular enzymes to degrade and utilize various types of organic substances in the ocean, including polysaccharides, amino acids, proteins, nucleic acids and lipids (Mayali et al., 2014), as well as indolent organic substances such as polycyclic aromatic hydrocarbons and urea (Gutierrez et al., 2013). This group was also the main degraders of transparent exopolymer particles (Taylor and Cunliffe, 2017) and chitins (Enke et al., 2018), both of which are major constituents of POC. *Pseudomonadales* has the capability to produce many extracellular hydrolases to hydrolyze POM (Qin et al., 2011) and high molecular weight dissolved organic carbon (Nelson and Carlson, 2012). As for archaea, *Halobacteriales* and *Methanosarcinales* were more frequently observed in the greater particles at the upper boundary. These two groups could catalyze the terminal step in the degradation of organic matter in anoxic environments where light was limiting (Oren and Oremland, 2000; Kendall and Boone, 2006), possibly following high hydrolytic activity of POC-attached microbes in the larger particles at the upper boundary of the twilight zone (Ivancic et al., 2018). *Methanosarcinales* could convert the produced small molecules (formic acid and acetic acids) to methane (Xia et al., 2016), thus accelerate the peptidoglycan and lipopolysaccharide biosynthesis.

At the lower boundary of the twilight zone, polymers and amino acids were used preferentially. This might be due to the presence of *Rhodobacterales*, which was the main degrader of polymers, such as transparent exopolymer particles (Taylor and Cunliffe, 2017) and chitins (Enke et al., 2018). As for amino acids, they were mainly derived from marine biodegradation, protein hydrolysis, extracellular excretion, and metabolites at all levels of the food chain, and are important components of marine organic nitrogen and organic carbon. They were preferred by microorganisms as highly available carbon sources and transported into cells with high affinity (Grzymski and Dussaq, 2012). Different microbial groups and their carbon utilization capabilities could help to make better use of the different carbon sources in the twilight zone. These metabolic profiles could be complemented with metatranscriptomic analysis and the isotopic tracing in future studies to augment the understanding of the complex carbon cycling pathways in the twilight zone.

In this study, in terms of microbial communities, higher similarities were observed between larger fractions (i.e., > 50 and > 10 μm), and between the euphotic layer and upper boundary as well; in terms of microbial metabolic activity, significant difference existed between the at the upper and lower boundary of the twilight zone. This might be attributed to the vertical shifts of the organic detritus at different depths. In addition to polymers as the major carbon source, carbohydrates and amino acids were preferentially used by microbial community at the upper and lower boundary, respectively. It should be noted that the types of carbon sources contained in microplate were limited, and only provide a glimpse of the carbon source preference, and might not able to match the real *in situ* carbon source compositions in the natural sampling locations. In the future, isotopic tracing experiments together with meta-transcriptomics would help to elucidate the real process of carbon source utilization by microbes occurred in the twilight zone.

## Data availability statement

The datasets presented in this study can be found in online repositories. The names of the repository/repositories and accession number(s) can be found at: NCBI, BioProject ID PRJNA755200.

## Author contributions

HJ and HBL conceived and designed the experiment and revised the manuscript. FW and HL performed the experiment and analyzed the data. HL wrote the first draft. All authors contributed to the article and approved the submitted version.

## Funding

This work was supported by the National Natural Science Foundation of China (41776147), the Training Program of the Major Research Plan of the National Natural Science Foundation of China (91751116), the National Key Research and Development Program of China (2016YFC0304905) and the Hainan Provincial Natural Science Foundation of China for High-level Talents (420RC677).

## Conflict of interest

The authors declare that the research was conducted in the absence of any commercial or financial relationships that could be construed as a potential conflict of interest.

## Publisher’s note

All claims expressed in this article are solely those of the authors and do not necessarily represent those of their affiliated organizations, or those of the publisher, the editors and the reviewers. Any product that may be evaluated in this article, or claim that may be made by its manufacturer, is not guaranteed or endorsed by the publisher.

## Footnotes

^1^ www.novogene.com

^2^ www.arb-silva.de
