# Supplementary material for: Metabolic activity and community structure of prokaryotes associated with particles in the twilight zone of the South China Sea
Source: Front Microbiol. 2022 Dec 6;13:1056860. doi: 10.3389/fmicb.2022.1056860 (PMC9763726; doi:10.3389/fmicb.2022.1056860)
Supplement: Supplementary file 1 [file Data_Sheet_1.docx]

**Supplementary Materials**

**Table S1.** The relative abundance of core and shared OTUs between different size-fractions of particles at each depth.

| Depth | > 50 μm | | | > 10 μm | | | > 1 μm | | |
| --- | --- | --- | --- | --- | --- | --- | --- | --- | --- |
| OTUs | Core | vs. > 10 μm | vs. > 1 μm | Core | vs. > 50 μm | vs. > 1 μm | Core | vs. > 50 μm | vs. > 10 μm |
| 0 m | 43.89% | 23.89% | 6.11% | 46.20% | 25.15% | 9.94% | 63.71% | 13.71% | 10.08% |
| 200 m | 22.60% | 22.12% | 6.01% | 27.01% | 26.44% | 7.76% | 51.37% | 14.75% | 16.39% |
| 800 m | 28.40% | 15.38% | 17.75% | 22.97% | 12.44% | 10.05% | 24.00% | 10.50% | 13.00% |

**Table S2.** The relative abundance of core and shared OTUs between different depths in different size-fraction of particles.

| Size-fraction | 0 m | | | 200 m | | | 800 m | | |
| --- | --- | --- | --- | --- | --- | --- | --- | --- | --- |
| OTUs | Core | vs. 200 m | vs. 800 m | Core | vs. 0 m | vs. 800 m | Core | vs. 0 m | vs. 200 m |
| > 50 μm | 17.50% | 26.11% | 16.67% | 15.14% | 22.60% | 9.13% | 37.28% | 8.28% | 22.49% |
| > 10 μm | 17.25% | 21.05% | 4.09% | 16.95% | 20.69% | 8.91% | 28.23% | 11.48% | 14.83% |
| > 1 μm | 16.94% | 12.90% | 9.68% | 22.95% | 17.49% | 22.40% | 21.00% | 11.50% | 20.50% |

**Figure legends:**

**Figure S1**. The abundance of 16S rRNA gene with different size-fraction particles in different water depths and the vertical distributions of prokaryotic cell abundance. The symbols of dark green, dark blue and dark purple of circles stand for prokarytoic (*Synechococcus* + *Prochlorococcus*) cell abundances with > 50, 10 and 1 μm size-fraction particles, respectively, while the symbols of dark green, dark blue and dark purple bar stand for > 50, 10 and 1 μm size-fraction particles, respectively.

**Figure S2**. Biplot of the redundancy analysis integrating environmental parameters and the microbial communities of different size-fraction particles at different water depths.

**Figure S3.** Venn diagrams representing the overlap of OTUs among total, 0, 200 and 800 m, and the microbial community composition at the order level for the shared OTUs among at 0, 200 and 800 m (A); the overlap of OTUs among total, > 50, 10 and 1 μm size-fraction particles and the microbial community composition at the order level for the shared OTUs among > 50, 10 and 1 μm size-fraction particles (B).

**Figure S4.** Potential metabolism categories of the core OTUs referred from KEGG metabolism modules with cluster at the relative abundance of functional gene level. * *p* < 0.05.

**Figure S5.** Utilization capability of the six major carbon groups, carbohydrates (A, B and C), amino acids (D, E and F), polymers (G, H and I), phenolic acids (J, K and L) and amine (M, N and O), carboxylic acids (P, Q and R), by microbes on different size-fraction particles at different water depths. The gray bar stands for the periode of the higher carbon sources utilization rate by microbes on different size-fraction particles at the upper and lower boundary of the twilight zone.

**Figure S6.** Microbial metabolic activity (A, B, and C), R (D, E, and F), D (H, I, and J), and H (K, L and M) associated with different size-fraction particles at different water depths during cultivation of 52 days.


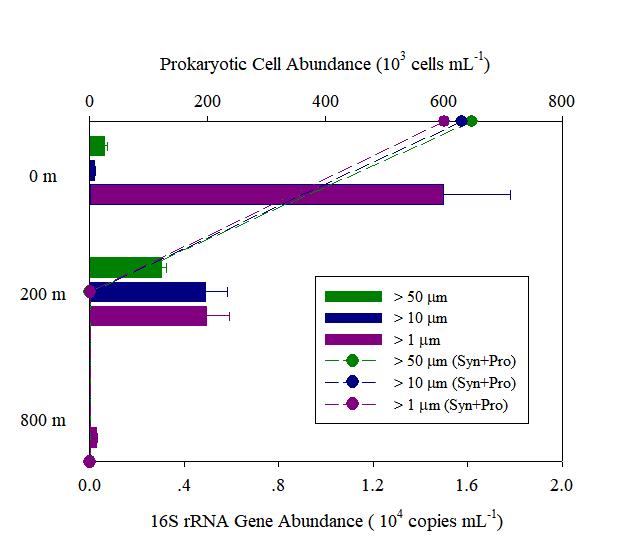


**Figure S1**


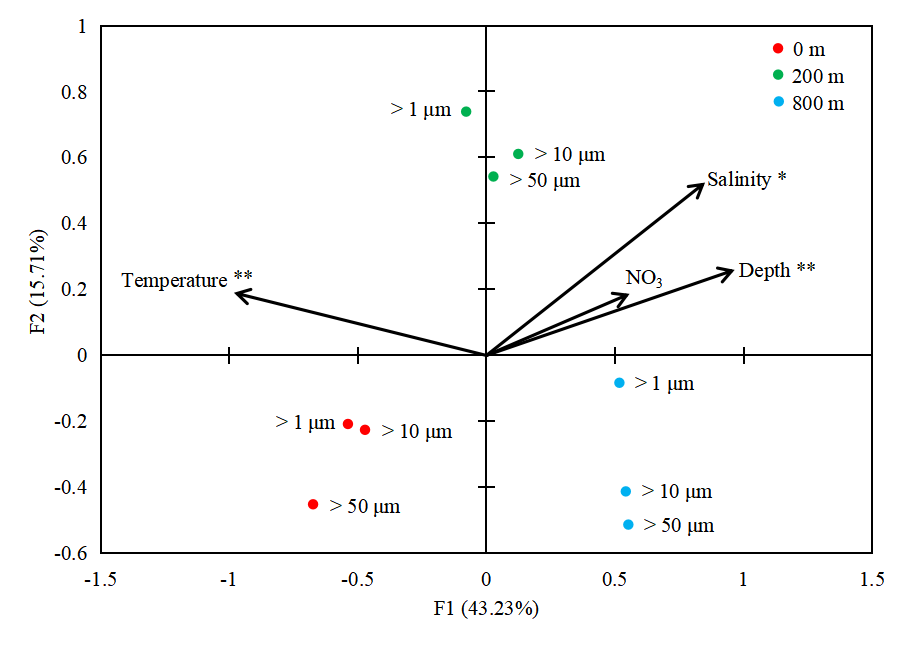


**Figure S2**


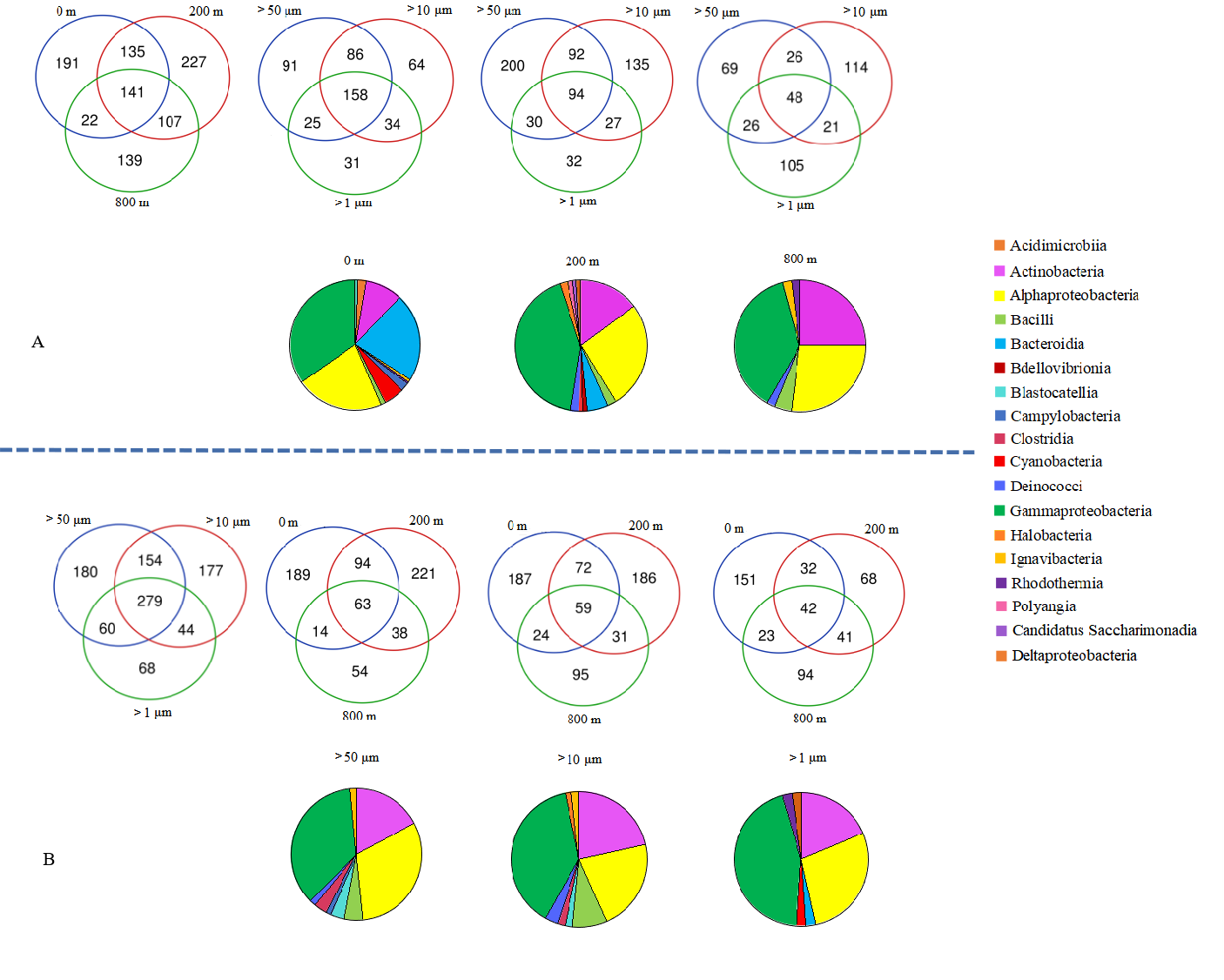


**Figure S3**


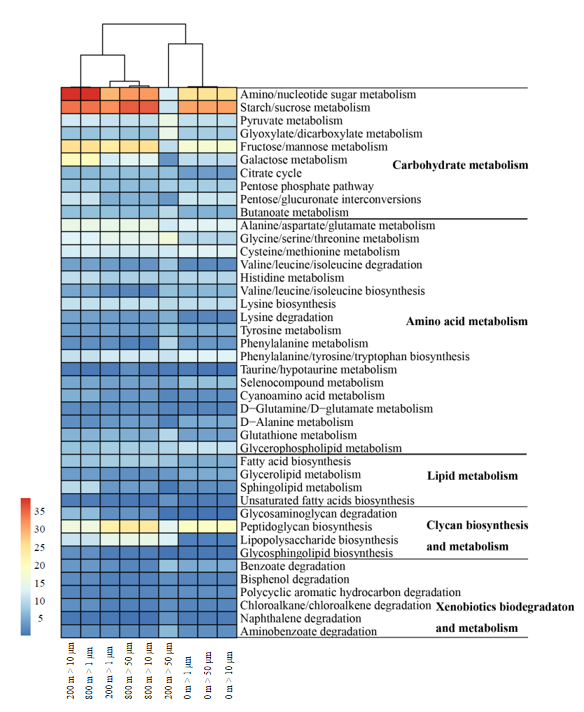


**Figure S4**


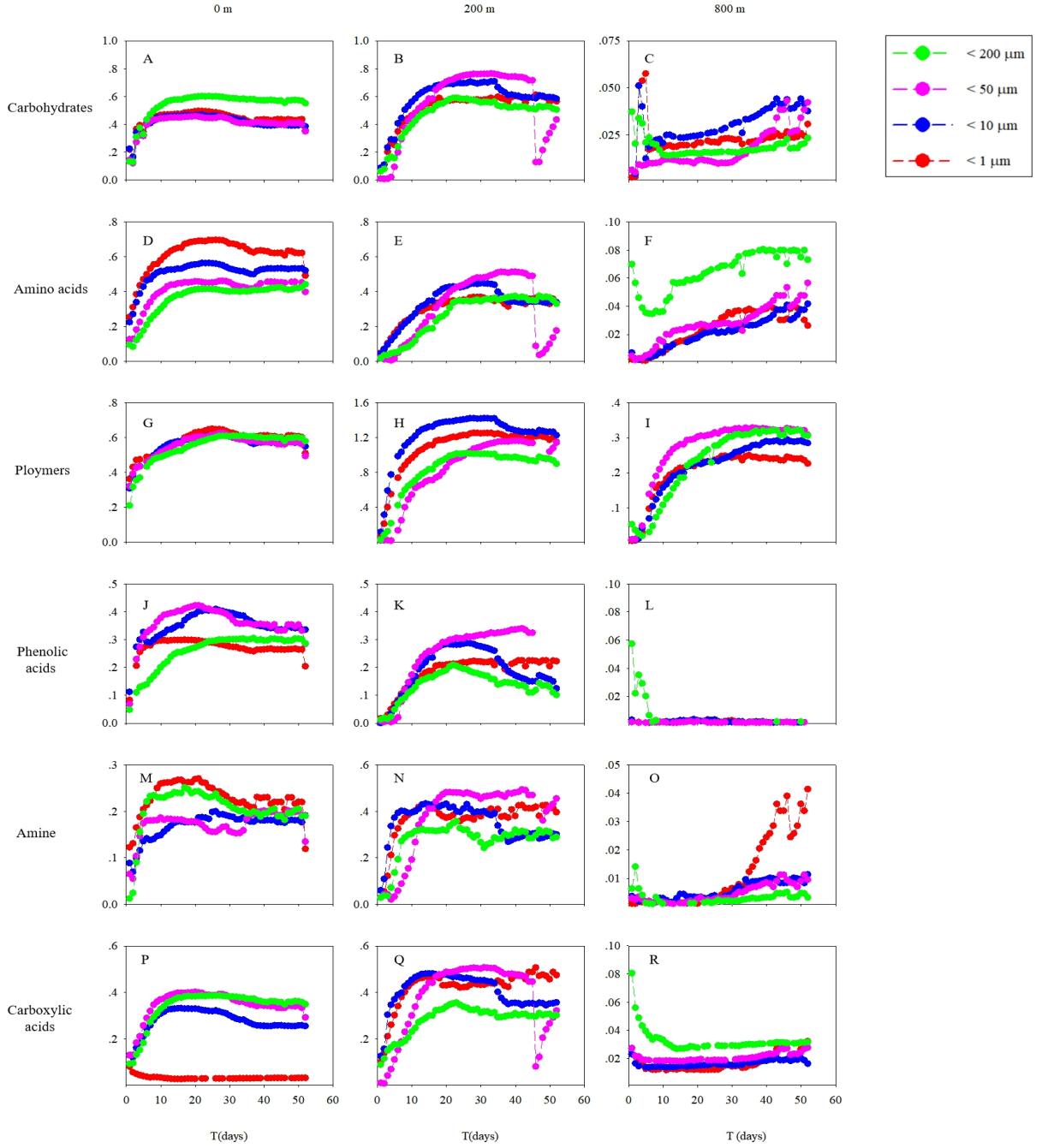


**Figure S5**


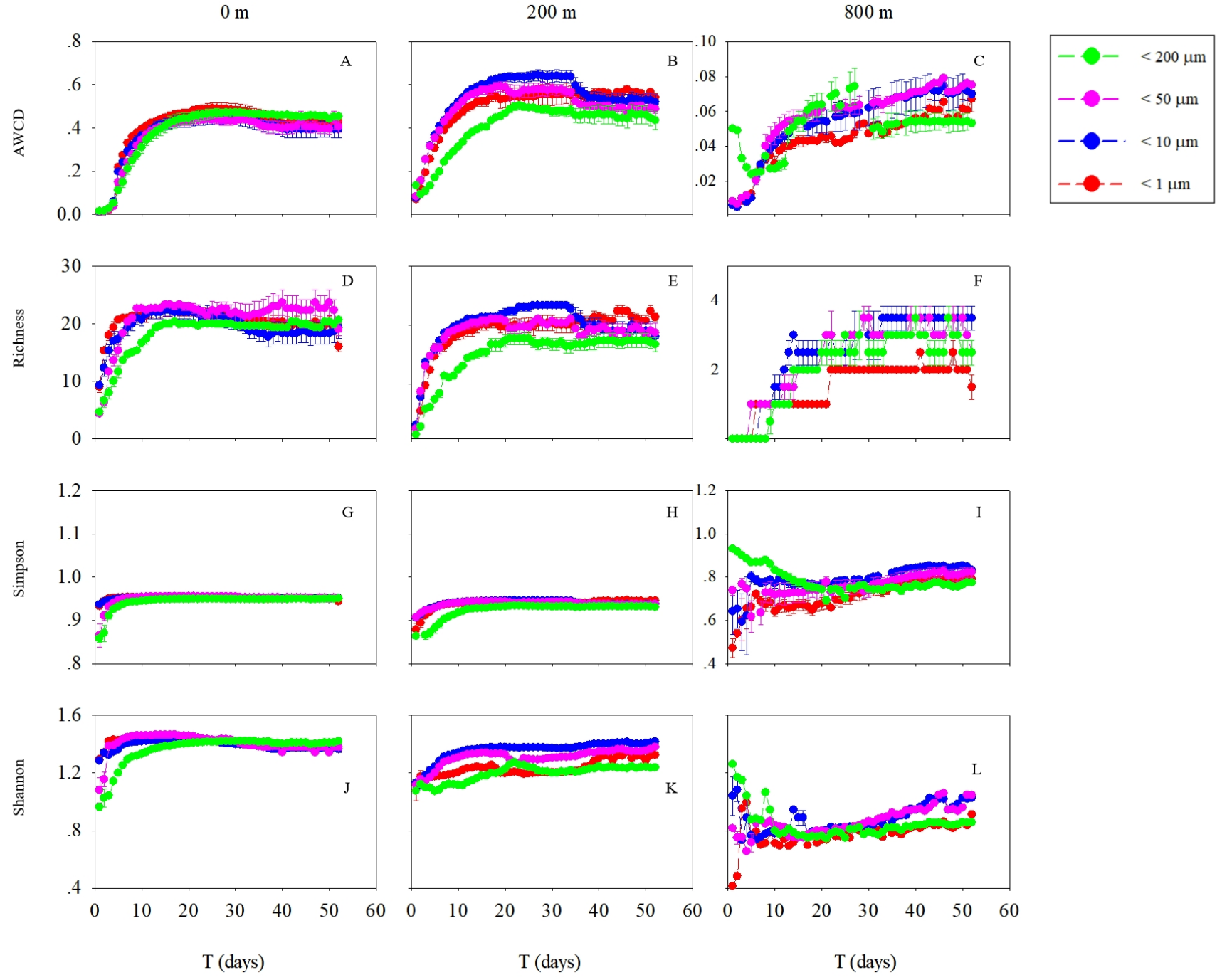


**Figure S6**
